# Effects of Mindfulness Based Cognitive Therapy (MBCT) and Compassion Focused Therapy (CFT) on Symptom Change, Mindfulness, Self-Compassion, and Rumination in Clients With Depression, Anxiety, and Stress

**DOI:** 10.3389/fpsyg.2019.01099

**Published:** 2019-05-17

**Authors:** Anna Dora Frostadottir, Dusana Dorjee

**Affiliations:** ^1^MSc in Mindfulness-Based Approaches, Director of the Mindfulness Centre in Reykjavik, Reykjavik, Iceland; ^2^Department of Education, Psychology in Education Research Centre, University of York, York, United Kingdom; ^3^School of Psychology, Bangor University, Bangor, Wales

**Keywords:** mindfulness, self-compassion, rumination, depression, anxiety, stress, MBCT, CFT

## Abstract

**Objectives:** Over the past decade there has been an increasing interest in exploring self-compassion as a related and complementary construct to mindfulness. Increases in self-compassion may predict clinical outcomes after MBCT and cultivation of compassion toward self and others is central to CFT. This pilot study compared the impact of MBCT applying implicit self-compassion instructions and CFT employing explicit self-compassion instructions on symptom change, mindfulness, self-compassion, and rumination.

**Method:** This non-randomized wait-list controlled study (*N* = 58) with two intervention arms (MBCT *N* = 20, CFT *N* = 18, Control *N* = 20) assessed the outcomes of clients with depression, anxiety, and stress symptoms from before to after the interventions and at one month follow up (MBCT *N* = 17, CFT *N* = 13, Control *N* = 13).

**Results:** Both treatments resulted in significant increases in mindfulness and self-compassion and decreases in rumination, depression, anxiety, and stress. Furthermore, MBCT enhanced mindfulness for people who were initially high in rumination, whereas CFT enhanced mindfulness across the board.

**Conclusion:** The findings suggest that both MBCT and CFT, and hence implicit or explicit self-compassion instructions, produce similar clinical outcomes with CFT enhancing mindfulness regardless of client's rumination level.

## Introduction

Mindfulness meditation practices are increasingly being incorporated into clinical treatments for a variety of mental health problems with positive results in reducing emotional distress and promoting psychological well-being (Hofman et al., 2010; Keng et al., 2011; Piet and Hougaard, 2011; Goyal et al., 2014). Mindfulness has its roots in Buddhism and is most often defined as “the awareness that emerges through paying attention on purpose, in the present moment, and non-judgmentally to the unfolding of experience moment by moment” (Kabat-Zinn, 2003, p. 145) in the secular therapeutic context. The two most widespread mindfulness-based programs are Mindfulness-Based Stress Reduction (MBSR; Kabat-Zinn, 1990) and Mindfulness-Based Cognitive Therapy (MBCT; Segal et al., 2002). The MBSR is intended for a wide range of clinical and non-clinical populations with promising cumulative evidence for its effects (Grossman et al., 2004; Chiesa and Serretti, 2009; Bohlmeijer et al., 2010). MBCT is based on MBSR and also integrates cognitive approach and instructions. It was developed as a relapse prevention for people with recurrent depression and has been found to reduce the risk of depression relapse by approximately half (Teasdale et al., 2000; Ma and Teasdale, 2004). It has also been shown effective for people dealing with anxiety, stress, irritability, and exhaustion (Hofman et al., 2010; Khoury et al., 2013).

Aside from mindfulness, another construct stemming from Buddhist psychology that is also being increasingly incorporated into meditation practices in clinical treatments is compassion toward self and others (Germer, 2009; Gilbert, 2009a; Neff, 2011). The standard definition of compassion is derived from the writings of the Dalai Lama (1995) who defined it as “a sensitivity to suffering in self and others with a commitment to try to alleviate and prevent it.” In the therapeutic context the construct of self-compassion is often used as defined by Kristin Neff (2015). According to Neff, self-compassion involves meeting ourselves with warmth and understanding when we suffer rather than ignoring our pain or criticizing ourselves—just as we would treat a good friend.

Research has shown that self-compassion is associated with decreased psychological distress and increased positive affect (Neff and Dahm, 2014). A recent meta-analysis by MacBeth and Gumley (2012) documented a large effect size for the relationship between greater self-compassion and lower levels of mental health problems such as depression, anxiety, and stress. More self-compassionate people also report lower levels of rumination (Neff, 2003a) and self-criticism (Ehret et al., 2015), which are known risk factors for depression and anxiety (Blatt, 1995; Nolen-Hoeksema, 2000); indicating that self-compassion might be a possible emotion regulation strategy for emotional difficulties.

In the context of MBSR and MBCT, the cultivation of self-compassion is implicit and an indirect intention of the programs. In fact, Segal et al. (2002) who developed MBCT advise mindfulness teachers against explicitly discussing or teaching self-compassion in the program. Rather, they suggest that participants learn the principles of self-compassion implicitly by the kind and compassionate embodiment of the teachers (Segal et al., 2013). Indeed, according to Germer and Barnhofer (2017) the second part of Kabat-Zinn's definition of mindfulness, non-judgemental acceptance, can be taken as indicating that compassion toward self and others and mindfulness are intrinsically linked. However, a recent study by Hildebrandt et al. (2017) explored the effects of three different 3-month mental training modules on self-compassion, The “Presence” module which aimed at cultivating present-moment-focused attention and body awareness, the “Affect” module which aimed at cultivating loving-kindness, gratitude and compassion and the “Perspective” module which aimed at cultivating meta-cognitive skills. Their findings indicated that present-moment mindfulness practices were not sufficient and only explicit socio-affective practices were able to increase self-compassion.

Contrary to that finding and despite the implicit nature in mindfulness practices in MBSR and MBCT, research has found that participation in MBSR (Bergen-Cico and Cheon, 2013) and MBCT (Rimes and Wingrove, 2011) leads to significant increases in both mindfulness and self-compassion. Looking closer into the mechanisms of self-compassion in MBCT, Kuyken et al. (2010) investigated the effects of MBCT compared to maintenance antidepressant medication (m-ADM) on mindfulness, self-compassion, cognitive reactivity, and relapse risk for depression. They found that increased mindfulness and self-compassion mediated the positive effect of MBCT on depressive symptoms at 15 months follow-up. They also found that cognitive reactivity was higher for MBCT participants compared to the m-ADM group, but only predicted poorer outcome for the m-ADM group, and not for the MBCT group. Further exploration of their findings indicated that increased self-compassion moderated and reduced the link between cognitive reactivity and relapse risk in the MBCT group, serving as an important protective factor. Based on this finding the second edition of the MBCT manual now explicitly states that MBCT aims to cultivate mindfulness and self-compassion (Segal et al., 2013).

In contrast to the implicit aspects of self-compassion in mindfulness practices in MBSR and MBCT, the practices of self-compassion meditation (Germer, 2009; Gilbert, 2009a; Neff, 2011) explicitly aim to cultivate compassion toward self and others. One approach incorporating explicit compassion instructions is Compassion Focused Therapy developed by Gilbert and colleagues (CFT; Gilbert, 2009a). CFT is an “integrated and multimodal approach that draws from evolutionary, social, developmental and Buddhist psychology, and neuroscience” (Gilbert, 2009b, p. 199). It was originally developed for people with long-term emotional problems, often associated with high levels of shame and self-criticism, and delivered as an individual therapy. The CFT seeks to help individuals develop compassion for self and others (Gilbert, 2017). It involves educating them about how the brain operates in terms of three types of emotion-regulation systems; the threat system, the drive system, and the soothing system. The CFT suggests that self-compassion deactivates the threat system and activates the self-soothing system (Gilbert and Irons, 2005). Although there are many overlapping features of CFT and MBCT, i.e., cultivating mindfulness, body awareness, and grounding mentalization training and the use of psychoeducation, it is very clear that MBCT puts the primary focus on cultivating mindfulness whereas CFT puts it on cultivating compassion toward self and others.

Although CFT for groups has not yet been manualized. Gilbert and Procter (2006) developed the first group-based version of CFT for patients with severe long-term difficulties in a cognitive-behavioral-based day center. Following 12 weekly sessions, they found significant reductions in depression, anxiety, self-criticism, shame, inferiority, and submissive behavior. Subsequent studies explored the use of group-based CFT in other clinical settings. Similar positive effects have also been found for people with psychosis (Laithwaite et al., 2009; Braehler et al., 2013), people with personality disorder (Lucre and Corten, 2013), people with eating disorders (Goss and Allan, 2010; Gale et al., 2012; Kelly et al., 2016) and people with long-term and severe mental health problems (Heriot-Maitland et al., 2014).

Due to the different instructions in mindfulness and self-compassion/compassion practices it is reasonable to assume that they have different impact on people (Germer, 2009). Researchers have begun to explore the question of “what for whom”, i.e., to match a practitioner with either mindfulness or compassion training. In relation to that, the findings from a study conducted by Barnhofer et al. (2010) suggested that those experiencing higher levels of rumination may benefit from meditations focusing on mindfulness, whereas people with lower levels of rumination may benefit more from loving kindness practices. We need to keep in mind that traditional Buddhist literature clearly distinguishes loving kindness and compassion (Wallace, 1999), however loving-kindness practices use explicit instructions to induce compassion toward self and others.

Cumulatively, the outlined evidence suggests that mindfulness-based programs, even though they cultivate self-compassion implicitly, are effective in reducing emotional distress in part *because* they cultivate self-compassion. There is also initial evidence that group CFT, a program targeting development of compassion toward self and others explicitly, can result in reductions in anxiety, depression, and other symptoms similar to the effects of MBCT, yet it is not clear if these effects are mediated by self-compassion. The current study aimed to directly compare the effects of implicit self-compassion training applied in MBCT and explicit self-compassion training employed in CFT on symptom change, mindfulness, self-compassion, and rumination. We predicted that both treatments would increase self-compassion and mindfulness as well as reduce symptoms and rumination, but gains in self-compassion would be greater after CFT in comparison to MBCT. If this later prediction was supported, this may indicate that mindfulness-based programs would benefit from incorporating explicit self-compassion practices into them. Furthermore, given the preliminary evidence suggesting that people respond differently to meditation practices based on their tendency to ruminate, we wanted to explore whether CFT and MBCT differentially impact on participants' outcomes depending on their baseline levels of rumination. It could be expected that participants with higher baseline rumination benefit more from MBCT and those with lower rumination from CFT.

## Methods

### Participants

The study was conducted in a residential rehabilitation and health clinic in Iceland, where clients usually stay for 4 weeks. The clinic offers a holistic approach in treating their clients with a variety of programs, e.g., psychoeducation, exercise, nutrition, acupuncture, and massage. It offers two kinds of psychotherapies, MBCT, and CFT. The participants were recruited through convenience sampling; the treatment participants were allocated to the intervention starting at the time they began their rehabilitation. Clients suffering from mild to moderate anxiety, depression and/or stress symptoms and able to complete the 4-week intervention were informed about the research during their orientation day. Up to 15 clients are typically included in each treatment group at the clinic and treatment participants were therefore allocated to two MBCT and two CFT groups. Complete pre-post data sets were obtained from 58 participants, 20 from the MBCT group, 18 from the CFT group, and 20 from the control group. A complete pre-post and follow-up data was obtained from 43 participants, 17 from the MBCT group, 13 from the CFT group, and 13 from the control group. Table 1 shows the distribution of gender and age across the three arms of the study.

**Table 1:** Demographic variables for participants in the three arms of the study.

	**MBCT**	**CFT**	**Controls**
**PRE-POST DATA (*****n*** **=** **58)**			
Total number, *n*	20	18	20
Gender, women: *n* (%)	19 (95%)	15 (83%)	17 (85%)
Age (in years): M (SD)	49 (11.05)	53 (10.20)	51 (9.26)
**PRE-POST & FOLLOW-UP DATA (*****n*** **=** **43)**			
Total number, *N*	17	13	13
Gender, women: *n* (%)	16 (94%)	10 (77%)	12 (92%)
Age (in years): M (SD)	50 (11.38)	52 (10.07)	49 (8.10)

After removing outliers the complete pre-post data for all four measures was from 54 participants, 19 from the MBCT group, 17 from the CFT group, and 18 from the control group. The complete pre-, post-, and follow-up data for all four measures was from 32 participants, 13 from the MBCT group, 12 from the CFT group, and 7 from the control group.

### Procedure

The study was approved by the ethics committee in the School of Psychology at Bangor University in the UK as well as from the Ethic Committee at the Directorate of Health in Iceland. Treatment participants were asked to complete the pre- and post-treatment questionnaires at the beginning of the first session and at the end of the last session in the MBCT and CFT groups. At the follow-up assessment 1 month after treatment participation, participants completed follow-up questionnaires electronically. Participants in the control group also completed the questionnaires at the same time points as treatment participants electronically.

The treatments were delivered by two experienced therapists, one of them is a clinical psychologist and the other is a psychiatric nurse. They have both been trained in MBCT at Bangor University and in CFT by Paul Gilbert. The clinical psychologist was also under Paul Gilberts' supervision. To ensure quality of the treatments they delivered, they had regular supervision sessions and provided each other with feedback in terms of adherence to the treatments. However, no other formal external examination was put into place to ensure the fidelity of the treatments.

*MBCT* was delivered following the treatment protocol (Segal et al., 2002) modified for four weeks: it included eight two-hour-long sessions over four consecutive weeks (two sessions per week). Session content included guided mindfulness practices (i.e., body scan, yoga, sitting meditations of breath, body, sound, thoughts, and exploring difficulties); enquiry into participants' experiences of these practices; homework review and teaching/discussion of cognitive skills modified for depression, anxiety, and stress. An adequate dose of MBCT was defined as participation in at least four of the eight MBCT group sessions as recommended (Segal et al., 2002).

*CFT* was delivered following the generic treatment outline (Gilbert, 2009a) which the clinical psychologist based on the MBCT manual (Segal et al., 2002) under Gilberts' supervision: it included eight 2-h long sessions over 4 consecutive weeks (two sessions per week). Session content included guided mindfulness, compassion, and self-compassion practices; enquiry into participants' experiences of these practices; review of homework; imagery; videos; experiential exercises; and teaching/discussion of the three interconnecting emotion-regulation systems. An adequate dose of CFT was defined as participation in at least four of the eight CFT group sessions.

The control group was offered to attend MBCT or CFT after the intervention groups completed their programs.

### Research Design

This pilot study followed a pre-post design with a control group and two intervention arms. The assessments were conducted at the baseline, after the 4-weeks long interventions and at 1 month follow up. Participants who were in the treatment groups either received the 4-week MBCT or the 4-week CFT.

### Measures

Five-Facets of Mindfulness (FFMQ; Baer et al., 2006) consists of 39 times, rated on a five-point Likert-scale, assessing the five facets of mindfulness of *Observing, Describing, Non-judging of inner experience, Acting with awareness, and Non-reactivity to inner experience*. Carmody and Baer (2008) reported that the FFMQ subscales are sensitive to change and have high internal consistency (α = 0.75–0.91).

Self-Compassion Scale (SCS; Neff, 2003b) consists of 26 items, rated on a five-point Likert-scale, assessing the positive and negative aspects of the three main factors of self-compassion: *Self-Kindness* vs. *Self-Judgement*; *Common Humanity* vs. *Isolation*; and *Mindfulness* vs. *Over Identification*. Birnie et al. (2010) reported high internal consistency for SCS subscales (α = 0.77–0.81) and overall high convergent and discriminant validity. This measure has been criticized because of its factor structure (Williams et al., 2014; Muris et al., 2016), however, it was chosen to provide comparative insights into the effect sizes of self-compassion. It is worthwhile to note its overlap with measuring mindfulness as it has a mindfulness subscale.

Reflection Rumination Questionnaire (RRQ: Trapnell and Campbell, 1999) consists of 12 items, rated on a five-point Likert-scale, assessing three aspects of rumination: *ruminative self-attention, the tendency to rehash, re-evaluate or dwell on past events or experiences*. Trapnell and Campbell (1999) reported high internal consistency of the RRQ (α = 0.94).

Depression Anxiety and Stress Scales—Short Form (DASS-21; Lovibond and Lovibond, 1995) evaluates the severity symptoms of Depression, Anxiety, and Stress. It consists of 21 items in which individuals are required to indicate the presence of a symptom over the past week from 0 (did not apply to me at all over the last week) to 3 (applied to me very much or most of the time over the past week). Henry and Crawford (2005) reported good discriminant and convergent validity for DASS-21 and high internal consistency for depression (α = 0.88), anxiety (α = 0.82), and stress (α = 0.90) scales.

To date, normative data has not been created for an Icelandic population for these measures. Therefore, all four questionnaires have been translated into Icelandic and then back-translated to confirm the accuracy of the initial translation. It was expected that Icelandic participants would respond in similar ways to Western participants for which the norms have been established.

### Data Analysis

All data analyses were conducted using the Statistical Package for the Social Science (SPSS), version 22.0 (SPPS Inc, Chicago, IL, USA). Extreme outliers were removed from the data and the non-extreme outliers were winsorized on the upper and lower bounds of the group (95%) on a measure by measure basis. Internal consistency was calculated for each measure. Between-group comparisons at the pre-treatment time for all the measures examined possible baseline differences. Correlations among the dependent measures at pre-treatment time were also investigated for expected convergent/divergent patterns. A 2 (time: pre, post) × 3 (Group, MBCT, CFT, Control) mixed ANOVA examined changes in the scores of the dependent measures from pre to post. A 3 (time: pre, post, follow-up) × 3 (group: MBCT, CFT, Control) mixed ANOVA with a smaller sample due to attrition at the latter two time points examined changes in the scores from pre, to post and follow-up. Significant interactions for all the measures were investigated further for predicted differences using paired-samples *t*-tests.

## Results

The DASS had high internal consistency for the Depression (α = 0.84), Anxiety (α = 0.77), and Stress (α = 0.79) subscales. The FFMQ had high internal consistency for the Observing (α = 0.77), Describing (α = 0.75), Acting with Awareness (α = 0.83), Nonjudging (α = 0.90), and Nonreacting (α = 0.73) subscales. The SCS had high internal consistency for the Self-Kindness (α = 0.82), Self-Judgement (α = 0.81), Common Humanity (α = 0.72), Isolation (α = 0.84), and Over-identifying (α = 0.77) subscales but poor consistency for Mindfulness (α = 0.66) subscale. The RRQ had high internal consistency (α = 0.91).

There were no significant differences between the groups at pre-treatment time on any of the dependent measures, all ps > 0.05

Correlations among the dependent measures at pre-treatment time are shown in Table 2. All the relationships were in the expected directions.

**Table 2:** Intercorrelations of outcome measures at pre-intervention time (*N* = 57).

**Variable**	**DASS total**	**DASS-D**	**DASS-A**	**DASS-S**	**FFMQ**	**SCS**	**RRQ**
DASS total		0.87^**^	0.81^**^	0.86^**^	−0.58^**^	−0.67^**^	0.56^**^
DASS-D			0.52^**^	0.63^**^	−0.51^**^	−0.63^**^	0.37^**^
DASS-A				0.57^**^	−0.42^**^	−0.47^**^	0.44^**^
DASS-S					−0.52^**^	−0.58^**^	0.63^**^
FFMQ						0.74^**^	−0.60^**^
SCS							−0.59^**^
RRQ							

*DASS total, total scores of Depression Anxiety and Stress Scales; DASS-D, scores for the Depression subscale of DASS; DASS-A, scores for the Anxiety subscale of DASS; DASS-S, scores for the Stress subscale of DASS; FFMQ, Five Facet Mindfulness Questionnaire; SCS, Self-Compassion Scale; RRQ, Reflection Rumination Questionnaire*.

** *p < 0.01*.

### Pre-post Evaluations

#### DASS

The main effect of time *F*_(1, 51)_ = 49.04, *p* < 0.001) and the main effect of group *F*(2,51) = 3.90, *p* < 0.03) were significant. The interaction of time by group *F*_(1, 51)_ = 6.67, *p* < 0.001) was also significant. As shown in Table 3, participants in the MBCT group had significantly lower post-treatment total DASS scores *t*_(18)_ = 5.72, *p* < 0.001, *d* = 1.65, 95% CI [9.92, 21.45], and significantly lower post-treatment scores on the depression *t*_(18)_ = 4.19, *p* < 0.001, *d* = 1.11, 95% CI [2.50, 7.50], anxiety *t*_(18)_ = 4.34, *p* < 0.001, *d* = 1.11, 95% CI [1.74, 5.00], and stress *t*_(18)_ = 6.62, *p* < 0.001, *d* = 2.06, 95% CI [4.99, 9.64] subscales of DASS. Participants in the CFT group also had significantly lower post-treatment total DASS scores *t*_(16)_ = 6.91, *p* < 0.001, *d* = 1.98, 95% CI [8.40, 15.84], and significantly lower post-treatment scores on the depression *t*_(16)_ = 6.53, *p* < 0.001, *d* = 1.59, 95% CI [3.14, 6.16], anxiety *t*_(16)_ = 3.05, *p* < 0.008, *d* = 0.96, 95% CI [0.57, 3.19], and stress *t*_(16)_ = 5.78, *p* < 0.001, *d* = 1.85, 95% CI [3.54, 7.64] subscales. No significant change was found in DASS scores in the control group (all *p*s > 0.05).

**Table 3:** Pre- and post-test scores for the MBCT, CFT, and control groups analyzed with paired-samples *t*-tests.

	**MBCT (*****N*** **=** **19)**		**CFT (*****N*** **=** **17)**		**Controls (*****N*** **=** **18)**	
**Outcome**	**Pretest M (SD)**	**Posttest M (SD)**	**Pretest M (SD)**	**Posttest M (SD)**	**Pretest M (SD)**	**Posttest M (SD)**
DASS total	26.74 (11.81)	11.05 (6.40)^**^	20.59 (6.88)	8.47 (5.23)^**^	23.44 (12.03)	20.39 (11.75)
Depression	9.11 (5.42)	4.11 (3.30)^**^	7.94 (3.36)	3.29 (2.59)^**^	9.33 (5.04)	8.00 (4.90)
Anxiety	5.68 (3.87)	2.32 (1.83)^**^	3.06 (2.44)	1.18 (1.29)^**^	5.17 (4.44)	4.11 (3.76)
Stress	11.95 (4.22)	4.63 (2.73)^**^	9.59 (3.32)	4.00 (2.69)^**^	8.94 (4.54)	8.28 (4.76)
FFMQ	111.47 (18.94)	127.05 (10.93)^**^	112.41 (14.33)	125.71 (13.34)^**^	119.50 (11.04)	119.83 (15.22)
SCS	71.42 (18.98)	82.74 (14.60)^**^	73.82 (13.60)	79.12 (13.87)^*^	75.00 (14.27)	75.56 (14.40)
RRQ	44.63 (10.70)	36.63 (7.41)^**^	44.82 (6.30)	39.76 (8.57)^**^	40.56 (9.12)	41.11 (10.05)

*M, mean; SD, standard deviation*.

T-test significant at

* p < 0.05 and

** *p < 0.001*.

#### FFMQ

The results revealed a significant main effect of time *F*_(1, 51)_ = 30.80, *p* < 0.001) and a significant main effect of group *F*_(2, 51)_ = 0.01, *p* > 0.05). The interaction of time by group *F*_(1, 51)_ = 7.40, *p* < 0.002) was also significant. As shown in Table 3, participants in the MBCT group had significantly higher post-treatment FFMQ scores *t*_(18)_ = −3.86, *p* < 0.001, *d* = −1.01, 95% CI [−24.05, −7.11]. Participants in the CFT group also had significantly higher post-treatment FFMQ scores *t*_(16)_ = −5.64, *p* < 0.001, *d* = −0.96, 95% CI [−18.30, −8.29]. No significant change was found in FFMQ scores in the control group (*p* > 0.05).

#### SCS

The main effect of time was significant *F*_(1, 51)_ = 17.95, *p* < 0.001) and the main effect of group was not significant *F*_(2, 51)_ = 0.08, *p* > 0.5). The interaction of time by group *F*_(1, 51)_ = 7.40, *p* < 0.002) was significant. As shown in Table 3, participants in the MBCT group had significantly higher post-treatment SCS scores *t*_(18)_ = −3.92, *p* < 0.001, *d* = −0.67, 95% CI [−17.38, −5.26]. Participants in the CFT group also had significantly higher post-treatment SCS scores *t*_(16)_ = −2.20, *p* < 0.05, *d* = −0.39, 95% CI [−10.40, −0.19]. No significant change was found in SCS scores in the control group (*p* > 0.05).

#### RRQ

**Figure 1:**
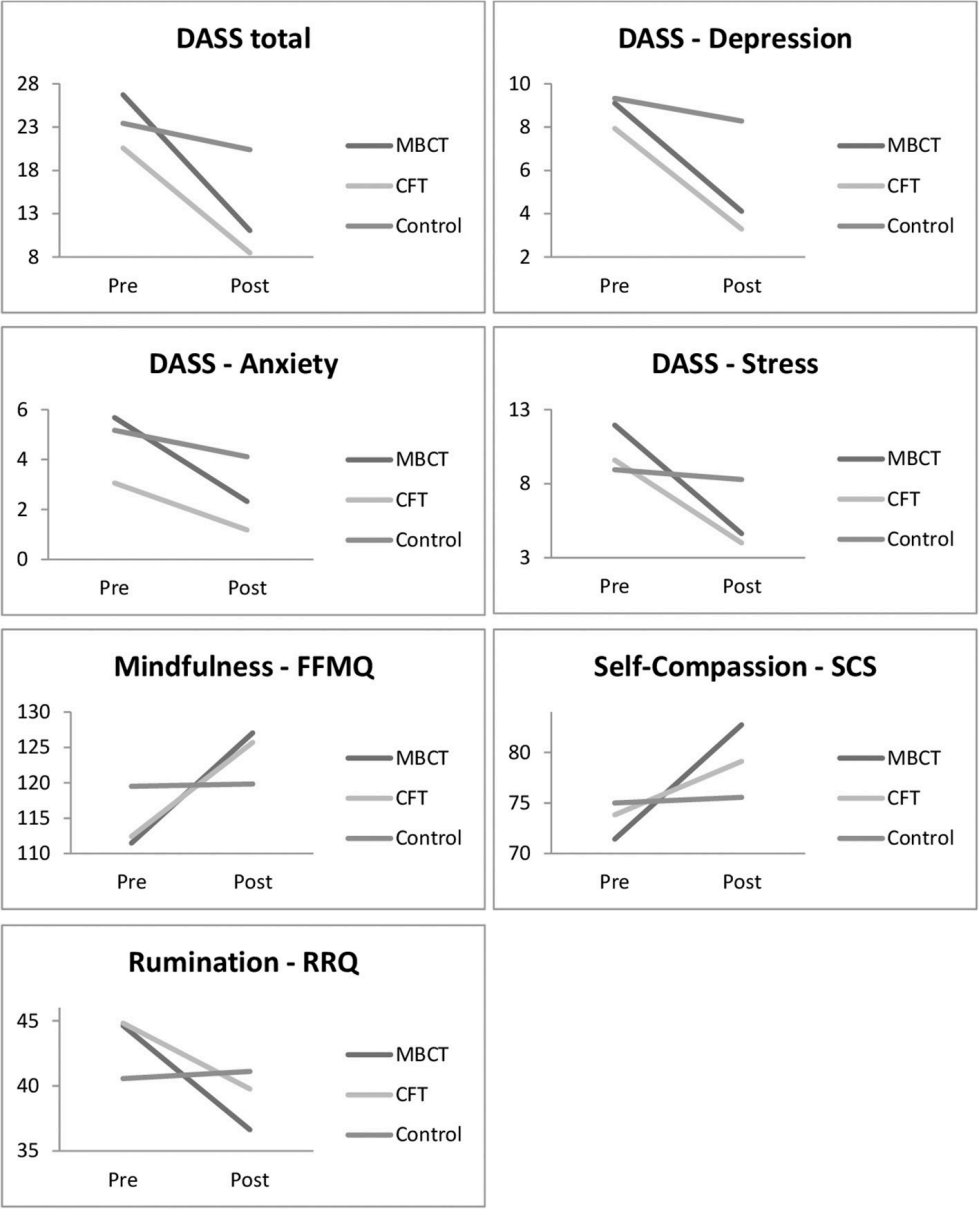
Changes in mean scores for all measures from pre to post-treatment in the MBCT, CFT, and Control groups.

The main effect of time was significant *F*_(1, 51)_ = 17.95, *p* < 0.001) and the main effect of group was not significant *F*_(2, 51)_ = 0.08, *p* > 0.5). The interaction of time by group *F*_(1, 51)_ = 7.40, *p* < 0.002) was significant. As shown in Table 3, participants in the MBCT group had significantly lower post-treatment RRQ scores *t*_(18)_ = 3.73, *p* < 0.002, *d* = 0.87, 95% CI [3.49, 12.51]. Participants in the CFT group also had significantly lower post-treatment RRQ scores *t*_(16)_ = 3.49, *p* < 0.003, *d* = 0.67, 95% CI [1.98, 8.14]. No significant change was found in RRQ scores in the control group (*p* > 0.05). The mean scores for pre- and post-treatment scores on the dependent measures for participants in the treatment groups and the control group are shown in Figure 1.

### Pre, Post, Follow-Up Evaluations

#### DASS

The main effect of time *F*_(2, 58)_ = 21.85, *p* < 0.001) and the main effect of group *F*_(2, 29)_ = 5.69, *p* < 0.01) were significant. The interaction of time by group *F*_(4, 58)_ = 6.20, *p* < 0.01) was also significant. As shown in Table 4, participants in the MBCT group had significantly lower post-treatment total DASS scores *t*_(12)_ = 5.26, *p* < 0.001, *d* = 1.86, 95% CI [9.77, 23.61], and lower post-treatment scores on the depression *t*_(12)_ = 4.09, *p* < 0.001, *d* = 1.35, 95% CI [2.77, 9.08], anxiety *t*_(12)_ = 4.16, *p* < 0.001, *d* = 1.39, 95% CI [1.79, 5.75], and stress *t*_(12)_ = 5.40, *p* < 0.001, *d* = 1.96, 95% CI [4.18, 9.82]. These reductions were maintained at follow-up as the participants had significantly lower follow-up total DASS scores than at pre-test *t*_(12)_ = 5.06, *p* < 0.001, *d* = 1.78, 95% CI [9.11, 22.89], and similarly significantly lower follow-up scores on the depression *t*_(12)_ = 3.96, *p* < 0.002, *d* = 1.35, 95% CI [2.76, 9.54], anxiety *t*_(12)_ = 3.83, *p* < 0.002, *d* = 1.39, 95% CI [1.66, 6.04], and stress *t*_(12)_ = 5.65, *p* < 0.000, *d* = 1.79, 95% CI [3.69, 8.31]. No significant change was found in the post to follow-up DASS scores in the MBCT group (*p* > 0.05). Participants in the CFT group had significantly lower post-treatment total DASS scores *t*_(11)_ = 4.76, *p* < 0.001, *d* = 1.60, 95% CI [5.82, 15.84], and significantly lower post-treatment scores on the depression *t*_(11)_ = 4.42, *p* < 0.001, *d* = 1.29, 95% CI [2.05, 6.12], and stress *t*_(11)_ = 4.28, *p* < 0.001, *d* = 1.64, 95% CI [2.59, 8.07] subscales. However, no significant change was found in their post-treatment scores on the anxiety subscale of DASS (*p* > 0.5). The reductions were maintained at follow-up as the participants in the CFT group had significantly lower follow-up total DASS scores in comparison to pre-treatment *t*_(11)_ = 3.03, *p* < 0.02, *d* = 1.41, 95% CI [2.32, 14.68], and similarly significantly lower follow-up scores on the depression *t*_(11)_ = 3.15, *p* < 0.01, *d* = 1.05, 95% CI [0.98, 5.52], and stress *t*_(11)_ = 3.01, *p* < 0.02, *d* = 1.22, 95% CI [1.03, 6.63]. However, no significant change was found from pre-treatment to follow-up scores on the anxiety subscale (*p* > 0.05). No significant changes were found in the post to follow-up DASS scores in the CFT group (all *ps* > 0.05). No significant changes were found in the pre, post and follow-up DASS scores in the control group (all *p*s > 0.05).

**Table 4:** Pretest, post-test, and follow-up mean scores for the MBCT, CFT, and control groups analyzed with paired sample *t*-tests.

	**MBCT (*****N*** **=** **13)**			**CFT (*****N*** **=** **12)**			**Controls (*****N*** **=** **7)**		
**Outcome**	**Pretest M (SD)**	**Posttest M (SD)**	**Follow-up M (SD)**	**Pretest M (SD)**	**Posttest M (SD)**	**Follow-up M (SD)**	**Pretest M (SD)**	**Posttest M (SD)**	**Follow-up M (SD)**
DASS total	29.23 (11.48)	12.54 (5.44)^**^	13.23 (5.43)^**^	19, 17 (7.30)	8.33 (6.24)^**^	10.67 (4.40)^*^	21.14 (10.85)	22.43 (6.71)	19.43 (6.05)
Depression	10.69 (5.41)	4.77 (3.00)^**^	4.54 (3.53)^*^	7.33 (3.47)	3.25 (2.80)^**^	4.08 (2.68)^*^	8.57 (5.29)	8.43 (3.74)	5.86 (3.24)
Anxiety	6.31 (3.43)	2.54 (1.71)^**^	2.46 (1.90)^*^	2.92 (2.54)	1.50 (1.38)	1.50 (1.68)	3.71 (3.35)	3.86 (3.13)	3.57 (2.76)
Stress	12.23 (4.38)	5.23 (2.52)^**^	6.23 (1.83) ^**^	8.92 (3.50)	3.58 (3.00)^**^	5.08 (2.75)^*^	8.86 (4.18)	10.14 (2.91)	10.00 (4.28)
FFMQ	108.92 (15.06)	125.69 (11.09)^**^	126.85 (10.29)^**^	114.92 (14.82)	126.83 (12.19)^**^	124.92 (9.34)^*^	115.71 (8.22)	110.14 (14.38)	111.71 (12.89)
SCS	66.77 (17.41)	81.69 (13.14)^*^	81.92 (13.25)^**^	76.42 (14.28)	81.92 (11.60)	84.75 (7.14)^*^	75.00 (16.29)	73.71 (14.42)	75.43 (13.25)
RRQ	46.77 (9.02)	36.54 (8.62)^**^	38.15 (5.84)^**^	43.08 (6.78)	38.25 (9.15)^*^	38.25 (6.97)^*^	45.14 (10.95)	44.57(10.53)	45.86 (8.26)

*M, mean; SD, standard deviation*.

T-test significant at

* p < 0.05 and

** *p < 0.001*.

#### FFMQ

The main effect of time was significant *F*_(2, 58)_ = 11.33, *p* < 0.001), but the main effect of group was not significant *F*_(2, 29)_ = 1.92, *p* > 0.5). The interaction of time by group *F*_(4, 58)_ = 7.14, *p* < 0.003) was significant. As shown in Table 4, participants in the MBCT group had significantly higher post-treatment FFMQ scores *t*_(12)_ = −14.19, *p* < 0.001, *d* = −1.27, 95% CI [−25.49, −8.05]. This increase was maintained at follow-up as the participants in the MBCT group had significantly higher follow-up FFMQ scores in comparison to pre-treatment *t*_(12)_ = −6.25, *p* < 0.000, *d* = −1.39, 95% CI [−24.17, −11.68]. No significant change was found between the post to follow-up FFMQ scores in the MBCT group (*p* > 0.05). Participants in the CFT group also had significantly higher post-treatment FFMQ scores *t*_(11)_ = −4.52, *p* < 0.001, *d* = −0.88, 95% CI [−17.72, −6.12]. This increase was maintained at follow-up as the participants in the CFT group had significantly higher follow-up FFMQ scores in comparison to pre-treatment *t*_(11)_ = −3.08, *p* < 0.02, *d* = −0.81, 95% CI [−17.15, −2.85]. No significant change was found between the post to follow-up FFMQ scores in the CFT group (*p* > 0.05). No significant change was found in the pre, post and follow-up FFMQ scores in the control group (all ps > 0.05).

#### SCS

The main effect of time was significant *F*_(2, 58)_ = 9.83, *p* < 0.004), but the main effect of group was not significant *F*_(2, 29)_ = 0.69, *p* > 0.5). The interaction of time by group *F*_(4, 58)_ = 3.64, *p* < 0.04) was significant. As shown in Table 4, participants in the MBCT group had significantly higher post-treatment SCS scores *t*_(12)_ = −4.08, *p* < 0.002, *d* = −0.97, 95% CI [−22.89, −6.96]. This increase was maintained at follow-up as the participants in the MBCT group had significantly higher follow-up SCS scores in comparison to pre-treatment *t*_(12)_ = −4.32, *p* < 0.001, *d* = −0.98, 95% CI [−22.79, −7.52]. No significant change was found between the post and follow-up SCS scores in the MBCT group (*p* > 0.05). Participants in the CFT did not have significantly higher post-treatment SCS scores (*p* > 0.05) but they had significantly higher follow-up SCS scores *t*_(11)_ = −2.66, *p* < 0.03, *d* = −0.74, 95% CI [−15.23, −1.44]. No significant change was found between the post and follow-up SCS scores in the CFT group (*p* > 0.05). No significant change was found in the pre, post, and follow-up SCS scores in the control group (all ps > 0.05).

#### RRQ

**Figure 2:**
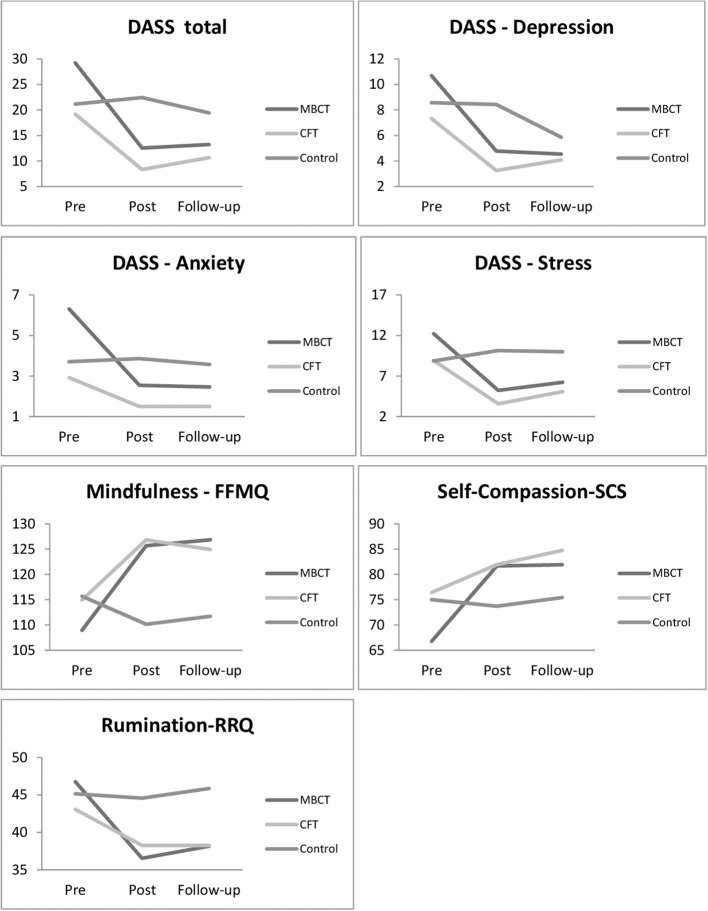
Changes in mean scores from pre- through post-treatment and to 1 month follow-up in the MBCT, CFT, and control groups.

he main effect of time was significant *F*_(2, 58)_ = 12.99, *p* < 0.001), but the main effect of group was not significant *F*_(2, 29)_ = 1.21, *p* > 0.5). The interaction of time by group *F*_(4, 58)_ = 4.13, *p* < 0.03) was significant. As shown in Table 4, participants in the MBCT group had significantly lower post-treatment RRQ scores *t*_(12)_ = 4.74, *p* < 0.001, *d* = 1.16, 95% CI [5.52, 14.94]. This reduction was maintained at follow-up as the participants in the MBCT group had significantly lower follow-up RRQ scores in comparison to pre-treatment *t*_(12)_ = 4.12, *p* < 0.001, *d* = 1.13, 95% CI [4.06, 13.17]. No significant change was found between the post and follow-up RRQ scores in the MBCT group (*p* > 0.05). Participants in the CFT group also had significantly lower post-treatment RRQ scores *t*_(11)_ = 2.61, *p* < 0.03, *d* = 0.60, 95% CI [0.76, 8.91]. This reduction was maintained at follow-up as the participants in the CFT group had significantly lower follow-up RRQ scores in comparison to pre-treatment *t*_(11)_ = 3.01, *p* < 0.02, *d* = 0.70, 95% CI [1.30, 8.36]. No significant change was found between the post and follow-up RRQ scores in the CFT group (*p* > 0.05). No significant change was found in the pre, post and follow-up RRQ scores in the control group (all ps > 0.05). The mean scores on the dependent measures across time for participants in the treatment groups and the control group are shown in Figure 2. In line with abovementioned results it shows that the effects of both treatments are mostly maintained at 1month follow-up.

### Impact of Pre-treatment Rumination Levels on the Treatment Outcomes

In order to investigate whether the tendency to ruminate affected how participants responded to the two interventions, the participants in the MBCT and in the CFT groups were further split into two subgroups based on a median split of their RRQ scores at pre-test. Higher than median scores determined higher rumination tendency and lower than median scores determined lower rumination tendency. This resulted in an additional factor of rumination which was included in the following analyses. A repeated-measures three-way ANOVAs with time (pre, post) as within-subject factor and intervention (MBCT, CFT, Control) and rumination (high, low) as between-subject factors, were conducted.

#### DASS

No significant three-way time × group × rumination interactions were found for either the total DASS scores or the DASS subscales scores (all *p*s > 0.05).

#### FFMQ

There was a significant three-way time × group × rumination interaction *F*_(1.48)_ = 8.65, *p* < 0.001). Participants in the MBCT group who were high in rumination had significantly higher post-treatment FFMQ scores *t*_(9)_ = −5.46, *p* < 0.001, *d* = −2.14, 95% CI [−38.75, −16.05], whereas the ones low in rumination did not show any significant change (*p* > 0.05). Participants in the CFT group who were high in rumination had significantly higher post-treatment FFMQ scores *t*_(8)_ = −4.11, *p* < 0.003, *d* = −1.21, 95% CI [−22.73, −6.38], and the ones low in rumination had also significantly higher post-treatment FFMQ scores *t*_(7)_ = −3.69, *p* < 0.008, *d* = −0.95, 95% CI [−19.48, −4.27]. Participants who were both high and low in rumination in the control group did not show any significant change in FFMQ scores (all *p*s > 0.05).

#### SCS

There was no significant three-way time x group x rumination interaction (*p* > 0.05).

## Discussion

This pilot study aimed to investigate the effects of MBCT applying implicit self-compassion instructions and CFT employing explicit self-compassion instructions on depression/anxiety/stress symptom change, mindfulness, self-compassion, and rumination. Participants in both treatment groups showed significant increases in mindfulness and self-compassion and decreases in rumination, depression, anxiety, and stress at post-test, whereas there were no changes reported for participants in the control group. The significant findings for the symptom change and mindfulness scores in the two treatment groups were accompanied by large effect sizes. The significant reductions in rumination were associated with large effect size in the MBCT group and medium effect size in the CFT group. Importantly, both participants in the CFT group and in the MBCT group showed significant improvements for the self-compassion scores at the post-test, with a medium effect size in the MBCT group and a small effect size in the CFT group. Furthermore, the exploration of the effects of MBCT and CFT on the participant outcomes depending on their pre-treatment tendency to ruminate revealed that MBCT participants who were high in rumination, but not those low in rumination, showed significantly increased mindfulness scores. Interestingly, for CFT participants mindfulness scores at post-test significantly increased in both rumination groups. However, all these significant findings need to be interpreted with caution given the non-randomized design of this study and a relatively small sample size.

In examining the effects of CFT and MBCT from pre-treatment to follow-up with a reduced sample of participants due to lack of response at this third time point (*N* = 32), participation in both treatments led to a significant decrease in total DASS and rumination scores and significant increase in mindfulness and self-compassion scores. However, only the MBCT group showed significant reductions in anxiety scores whereas the CFT group did not manifest a significant change.

Overall, findings of the current study are consistent with a number of studies that have shown that MBCT and CFT are effective at enhancing mindfulness and self-compassion and at reducing depression, anxiety, stress, and rumination (Teasdale et al., 2000; Ma and Teasdale, 2004; Gilbert and Procter, 2006; Gilbert, 2009a; Goss and Allan, 2010; Kuyken et al., 2010; Lowens, 2010; Rimes and Wingrove, 2011). This is the first study that has directly compared the effects of MBCT and CFT and according to our findings there were no significant differences in their effects, except for follow up reductions in anxiety with significant change in the MBCT group only. Interestingly, both MBCT and CFT resulted in significant improvements for self-compassion with a medium effect size for the MBCT group and small effect size in the CFT group which is contrary to the prediction that explicit cultivation of self-compassion leads to larger enhancements (Gilbert, 2009a).

Our findings partly support the notion that people differ in their response to the treatments based on their tendency to ruminate as participants who were high in rumination at pre-test in the MBCT group showed a significant increase in mindfulness at post-test and those with low rumination did not show improvements. Such selective increase in mindfulness for the high rumination participants lends further support to previous results with recurrently depressed participants in remission where only those with high rumination positively responded to a brief mindfulness induction (Barnhofer et al., 2010). This interesting finding is more broadly in line with the pervious evidence that MBCT is particularly effective for people with depression (Teasdale et al., 2000; Ma and Teasdale, 2004) as rumination is considered to be one of the key risk factors for depression (Nolen-Hoeksema, 2000). However, findings from the current study also surprisingly revealed that both high and low rumination participants in the CFT group showed significant increases in mindfulness at post-test which is contrary to Barnhofer's study (2010) where only participants with low rumination benefited from brief loving kindness instructions. Importantly, we haven't found differences between the high and low rumination groups for either MBCT or CFT in symptom change and self-compassion scores which somewhat limits the findings of differences in mindfulness gains and requires further investigation.

### Limitations

Overall, interpretation of the findings of the current study needs to take into account several limitations. First, the study did not employ a randomized controlled design. While this raises some concerns with regard to comparability of the groups, there were no significant differences found in any of the measures between the groups at pre-treatment. Second, the treatment groups were in a residential rehabilitation and health clinic during the treatment period, in which they were offered variety of other beneficial programs along with MBCT and CFT. This could have confounded the findings to some extent. Third, there is also the possibility that the findings of this study have been impacted by the small sample size and therefore need to be interpreted with caution (even though the combination of significant findings and large/medium effect sizes is reassuring). Shorter time-frame for delivery of MBCT and CFT (half in comparison to the usual 8-week format, yet equal number of sessions was covered) may have also impacted the results since it may take time to develop mindfulness and self-compassion skills. In addition, difficulties may arise with replicating this study with a larger sample, as CFT is not manualized like MBCT. In addition, no external examination was put into place in order to ensure the fidelity of the treatments. Finally, the follow-up time in the current study was limited to one month which does not allow for informed conclusions about possible longer-term effects of MBCT and CFT and their comparison.

Future studies should employ a randomized controlled design with a larger sample, in both clinical and non-clinical settings and with longer and repeated follow-ups. If conducted in a residential rehabilitation clinic, it would be recommended to compare the treatment groups to a control group that is also in the clinic at the same time but not receiving MBCT or CFT. It would be interesting to compare MBCT to a standardized manualized self-compassion program. Future studies could also include assessments such as behavioral or psychophysiological markers to bypass some of the limitations of self-reports. Impact of individual differences at the baseline, such as levels of rumination, on treatment outcomes needs further investigation.

In conclusion, the findings from the current study suggest that both MBCT and CFT are effective at enhancing mindfulness and self-compassion and at reducing depression, anxiety, stress, and rumination in clients with anxiety, depression, and stress difficulties. It seems that the implicit way of cultivating self-compassion in MBCT is just as effective as cultivating self-compassion explicitly in CFT. Furthermore, our findings partly support the hypothesis that participants differ in their response to meditation practices and that MBCT may be more effective at enhancing mindfulness for people who are high in rumination. Surprisingly, we have found that CFT may be effective at enhancing mindfulness for both those with high and low rumination. Further research is needed to conclusively elucidate the similarities and differences between effects of implicit and explicit self-compassion instruction on participant outcomes.

## Ethics Statement

The study was approved by the Research Ethics &amp; Governance Committee at Bangor University and the Ethic Committee at the Directorate of Health in Iceland.

## Author Contributions

AF designed and executed the study, conducted the data analyses, and wrote the manuscript. DD guided and supervised the design/execution of the study and data analyses, she edited the manuscript.

### Conflict of Interest Statement

The authors declare that the research was conducted in the absence of any commercial or financial relationships that could be construed as a potential conflict of interest.
